# Supplementary material for: Evaluation method on seismic risk of substation in strong earthquake area
Source: PLoS One. 2021 Dec 2;16(12):e0258792. doi: 10.1371/journal.pone.0258792 (PMC8639099; doi:10.1371/journal.pone.0258792)
Supplement: Attachment — Submitted filename: PONE-D-21-17828.pdf [file pone.0258792.s001.pdf]

PONE-D-21-17828

Rapid evaluation method on seismic damage state of substation in strong earthquake area

PLOS ONE

Review Comments: This paper presents a methodology to assess the damage state of electrical substations after a seismic event provided that the vulnerability curve of the substation for different damage states is already developed. The approach seems good but the followings should be checked and modified:

The authors claim the method as rapid evaluation, but it's not like that as the vulnerability curve should be developed at first which includes incremental dynamic analysis (IDA), development of PSDM, application of reliability theory for the development of substation vulnerability curve from elemental vulnerability.

The manuscript should be checked thoroughly to modify several errors. These include-

a) Tables and Figures should be always addressed and described in text before they appear (e.g. Fig. 8, 9, 10 are not addressed or described in text).
b) Table No. and Figure No. described in text are not matched with actual Table No. and Figure No., respectively (e.g. it is described in text that Table 9 represents Records of PGA, but it is actually in Table 7; actually Figure 14 represents Magnitude-Frequency Curve while in text it is wrongly mentioned Figure 13.).
c) Reference list contains many styles that should be unified and some references should be checked, e.g. in line 174 though Baker is not included in the author list Lin et al. 2011, why author says "Baker recommended)?

Abstract:

As PGA depends not only on distance but also on soil significantly, interpolation for PGA attenuation will not give good or reliable results.

In the flowchart (Fig. 2), as the materials, geometric parameters, etc. are collected for the selected single facility; so, algorithm should be "single power facility > materials, geometric parameters > FEM....".

<u>(3.1.1)</u>

a) Is there any reference for the design manual mentioned in line 139?
b) In Table 1, Number 6- Poisson's ratio- It should not be in Pa rather unitless.
c) For finite element modeling seismic damage characteristics is not required, only geometric and material properties are needed (line 139-140).
d) It will be more suitable to define the four vulnerability parts as key vulnerability parameters (e.g. top displacement of high-voltage bushing, max principal stress at the root of medium-voltage bushing).
e) In Table 2 (which must be addressed in text), it seems that the damage limit states are defined as reverse. micro cracks are defined for complete damage while parts separated and bushing broke off completely are defined for slight damage.
f) As the height of medium-voltage bushing is significantly smaller than the high-voltage bushing, why same displacement limit (e.g. 600 mm for complete damage) is defined for two?

<u>(3.1.2)</u>

a. In Table 4, parameters are not defined clearly, e.g. attribute value 0.7019 refers to characteristic .....(what)?
   [may be characteristic period, found later in line 197]
b. It should be clearly mentioned why earthuakes with very low PGA value (0.0180g - 0.0597g) are selected for IDA.
   What is the type of magnitude of the earthquakes (Mw, Ms...)?

PONE-D-21-17828

Rapid evaluation method on seismic damage state of substation in strong earthquake area

PLOS ONE

(3.1.3)

a) In line 197, it is mentioned that the amplitude adjustment ratios are 0.1g, 0.2g, 0.3g,....,1.5g. Is it amplitude adjustment ratios or amplitudes?
b) In Fig. 4, what is meant by [Ug/m], [Sg/MPa], [Uz/m], [Sz/m]?
c) In Fig. 4c, why >0.2m is marked while the limit is defined as 0.6m (in Fig. 4a >0.6m is marked for same limit state value)?

(3.1.4)

a) Parameters in equation 4 should be defined clearly
b) In line 229, though there is no section as 3.3.2 it mentioned as... simulation results from 3.3.2.

(3.2.1)

c) There is no description about the experts evaluation. The importance weights in Table 6 (addressed as Table 8 in text) should be checked.

(3.2.2)

d) What parameters are considered for nine different facilities and What are the limit state for those vulnerable parameters for different damage states?

(4.1)

e) The magnitude of 2008 Wuqia earthquake (Richter M 6.8) should be converted to Ms value as in the attenuation equation (eqn 1), it is denoted that magnitude should be in Ms.

(4.2.1)

a. In equation 10, N(>=M) denotes, rate of earthquake i.e. number of earthquakes per year with magnitude greater or equal M. This should be described correctly and also must be applied in the development of Magnitude-Frequency relationship.
b. The completeness of data can be checked by the plot of Cumulative No. of earthquakes Vs Time (Year) as per the simplified approach by Mulargia et al. (1987), or even by the method of Stepp (1972).
c. In the Magnitude-Frequency curve, vertical axis (earthquake frequency) should be plotted in log scale to represent the linear relationship.
d. It is not clear why the occurrence rate or frequency of maximum magnitude earthquake is 1, i.e. No. of earthquake per year is 1 [N(>=M)=1].

In conclusion it can be suggested that the article should be modified with application of recalculated values of various parameters as well as should be checked thoroughly to improve the write up.

Authors may consult with the following papers to improve the current state of the manuscript:

1. Hassanzadeh, R., Nedović-Budić, Z., Razavi, A. A., Norouzzadeh, M., & Hodhodkian, H. (2013). Interactive approach for GIS-based earthquake scenario development and resource estimation (Karmania hazard model). *Computers & geosciences*, *51*, 324-338.

2. Okazaki, K., Villacis, C., Cardona, C., Kaneko, F., Shaw, R., Sun, J., ... & Tobin, L. T. (2000). RADIUS: Risk assessment tools for diagnosis of urban areas against seismic disasters. In *RADIUS: Risk assessment tools for diagnosis of urban areas against seismic disasters* (pp. 38-38).

3. Mazumder, R. K., & Salman, A. M. (2019). Seismic damage assessment using RADIUS and GIS: A case study of Sylhet City, Bangladesh. *International journal of disaster risk reduction*, *34*, 243-254.

4. Applied Technology Council (ATC), ATC-13: Earthquake Damage Evaluation Data for California, *Applied Technology Council*, Washington, DC., 1985.
