# Supplementary material for: Evaluation method on seismic risk of substation in strong earthquake area
Source: PLoS One. 2021 Dec 2;16(12):e0258792. doi: 10.1371/journal.pone.0258792 (PMC8639099; doi:10.1371/journal.pone.0258792)
Supplement: Attachment — Submitted filename: Response to Reviewer and Eiditors.docx [file pone.0258792.s002.docx]

List of Responses

**Dear Editors and Reviewers:**

Thank you for your letter and for the reviewers’ comments concerning our manuscript entitled “Rapid evaluation method on seismic damage state of substation in strong earthquake area” (which has been modified to ‘Evaluation method on seismic risk of substation in strong earthquake area’) (ID: PONE-D-21-17828). Those comments are all valuable and very helpful for revising and improving our paper, as well as the important guiding significance to our researches. We have studied comments carefully and have made correction which we hope meet with approval. Revised portion are marked in the paper. The main corrections in the paper and the responds to the reviewer’s comments are as following:

**Responses to the editors:**

Thank you for your letter and suggestions. According to the comments, we have made a comprehensive revision to the text and submitted the missing documents. It includes that: 1) The style and format of the text have been modified according to the template and requirements of the magazine; 2) Improved the sharpness of most figures; 3) Improved the English writing level of the text; 4) the text was revised according to the reviewers’ comments; 5) The owner of Fig 1 in the text is one of the authors (Yongfeng Cheng), we have submitted the document of the source of the picture; 6) A data availability statement, a funding statement, and the source and rightsholder information have been submitted. 7) Our data was submitted to a public database. DOI <https://doi.org/10.5061/dryad.8gtht76q6>. In addition, we would like to add two authors Yongfeng Cheng and Sheng Li come from China Electric Power Research Institute They provided the following assistance in writing the text: 1) provide the data for the study; 2) provide the technical guidance; 3) the zoning and importance weights of power facilities are derived from the design manual and enterprise standards of China Electric Power Research Institute. Therefore, they are also one of the authors of the article. We hope we can get your approval.

Looking forward to hearing from you again!

**Reviewer#1:**

**1:** The authors claim the method as rapid evaluation, but it's not like that as the vulnerability curve should be developed at first which includes incremental dynamic analysis (IDA), development of PSDM, application of reliability theory for the development of substation vulnerability curve from elemental vulnerability

**Response:** Thanks to you for your suggestions and opinions Considering the comments, we think that this method is more inclined to the seismic risk assessment of substations in the region, rather than the rapid assessment of earthquake damage state. The method proposed in this paper is to build the vulnerability model of each voltage level substation in advance and form a database. After the earthquake, the risk of the substation will be evaluated by matching the ground motion evaluation results with the vulnerability model. We have modified the title ‘Rapid evaluation method on seismic damage state of substation in strong earthquake area’ to ‘Evaluation method on seismic risk of substation in strong earthquake area’ and the presentation ‘rapid evaluation of seismic damage’ to ‘evaluation of seismic risk’ in the text to better match the proposed method.

**2:** Tables and Figures should be always addressed and described in text before they appear (e.g. Fig. 8, 9, 10 are not addressed or described in text).

**Response:** We are very sorry for our incorrect writing. We have adjusted the position of Tables and Figures (include Table 2) and added the description of Figures (include Fig 4, 5, 8, 9, 10). Ensures that all Tables and Figures are described in text before they appear. **(Fig 4: line 215-2229)** These figures indicate that the seismic response values of four key vulnerability parameters ($U_{g}$, $S_{g}$, $U_{z}$, $S_{z}$) under 20 sets of seismic waves, where the red line is the median of the seismic response values of 20 seismic waves under different PGA (0.1 g, 0.2 g, …, 1.5g). The bushing comprises a brittle material, and shows no evident deformation. Therefore, the shaded part in the figure indicates that the bushing has been completely destroyed. **(Fig 5: line 252-261)** These figures illustrate that the probability of four seismic states of key vulnerability parameters under different PGA. It can be seen that the seismic vulnerability of high-voltage busing is higher than that of medium-voltage busing. High-voltage busing has a larger size than the medium, resulting in lower stiffness. As a result, high-voltage busing is more vulnerable to earthquakes. This shows the correctness of the analysis results from the side. **(Fig 8,9,10: line 309-320)** It can be seen that these seismic vulnerability models can be used to construct the relationship between different ground motion intensities and seismic damage states of substations in a probabilistic way. Then, the seismic risk of substations within the affected area can be quickly assessed after the earthquake.

**3:** Table No. and Figure No. described in text are not matched with actual Table No. and Figure No., respectively (e.g. it is described in text that Table 9 represents Records of PGA, but it is actually in Table 7; actually Figure 14 represents Magnitude-Frequency Curve while in text it is wrongly mentioned Figure 13.)

**Response:** We are very sorry for our carelessness in the writing The No. and descriptions of the Figures and Tables in text have been erratum.

**4:** Reference list contains many styles that should be unified and some references should be checked, e.g. in line 174 though Baker is not included in the author list Lin et al. 2011, why author says "Baker recommended)

**Response:** We are sorry that an error occurred when writing the reference, which has been revised. In fact, the first author of this reference is Baker JW. In addition, the style of the references has been unified.？

(Line 502-503): [27] Baker, J W, Lin T, Shahi S K, Jayaram N. New ground motion selection procedures and selected motions for the PEER transportation research program. Pacific Earthquake Engineering Research Center, 2011.

**Reviewer#2:**

**1:** As PGA depends not only on distance but also on soil significantly, interpolation for PGA attenuation will not give good or reliable results.

**Response:** Thanks to you for your suggestions and opinions. We did not consider the effects of site conditions in our previous studies. Therefore, this effect has been reconsidered and discussed in the text. We added a discussion on why site conditions are not considered and explain the impact of that on the evaluation results. (Line 98-106): However, these substations do not necessarily coincide with seismic observation stations network. In order to obtain the PGA of the substation location after the earthquake, the distribution law of PGA can only be obtained by fitting the PGA data of the seismic network. In general, the attenuation model of PGA is difficult to consider the effect of site conditions. It is a complex process to clarify the influence of site conditions of hundreds of substations in a region on PGA in detail. In general, as with seismic observation stations, the foundation of the substation is reinforced and can be thought of as free ground shaking. The effect of ignoring the amplifying effects of soft soils can be considered small. Therefore, ignoring site conditions can ensure the efficiency of seismic risk assessment of substations, and will not affect the accuracy of probability-based assessment

**2:** In the flowchart (Fig. 2), as the materials, geometric parameters, etc. are collected for the selected single facility; so, algorithm should be "single power facility > materials, geometric parameters > FEM.....

**Response:** Thank you for your guidance. The flowchart (Fig 2) has been modified.

Fig 2. Flowchart of seismic damage assessment of substation

**(3.1.1)**

**3:** Is there any reference for the design manual mentioned in line 139

**Response:** This design manual was published by State Grid Corporation of China. Information about the book was added to the references.

(Line 494-495): [23] State Grid Corporation of China. General design of power transmission and transformation project of State Grid. Beijing, China: China Electric Power Press. 2017.

**4:** For finite element modeling seismic damage characteristics is not required, only geometric and material properties are needed (line 139-140).

**Response:** Considering the reviewer’s suggestion, the relevant expressions in text have been modified according to the advice of experts. (Line 151-152): Then, the basic form of the finite element model is determined by extracting its geometric and material properties (Fig 3).

**5:** It will be more suitable to define the four vulnerability parts as key vulnerability parameters (e.g. top displacement of high-voltage bushing, max principal stress at the root of medium-voltage bushing).

**Response:** Thanks for your suggestion. In the full text, 'key vulnerable Parts' has been changed to' key vulnerable parameter '.

**6:** In Table 2 (which must be addressed in text), it seems that the damage limit states are defined as reverse. micro cracks are defined for complete damage while parts separated and bushing broke off completely are defined for slight damage.

**Response:** We are very sorry for our incorrect writing. The seismic damage state of power facilities defined in Table 2 is based on the enterprise standard, which is ‘Guidelines for seismic safety risk assessment of electrical substations (converter stations)’, issued by the State Grid Corporation of China. Table 2 has been errata and added references.

(Line177) Table 2. Four limit states of 750 kV main transformer

| Limit state | Define |
| --- | --- |
| Complete damage | Impaired major functions. It may take a long time (e.g., 60 days) to repair |
| Extensive damage | Partial function interruption. It can be repaired over a period of time (e.g., 15 days) |
| Moderate damage | Some functions can be restored after a short interruption |
| Slight damage | Function play without interruption |

(Line 498-499): [25] State Grid Corporation of China. Guidelines for seismic safety risk assessment of electrical substations (converter stations). Beijing, China: China Electric Power Press. 2021.

**7:** As the height of medium-voltage bushing is significantly smaller than the high-voltage bushing, why same displacement limit (e.g. 600 mm for complete damage) is defined for two?

**Response:** We are very sorry for our incorrect writing. The displacement of the top medium-voltage bushing should be 200mm for complete damage. 200mm was also used in subsequent analysis. So, errata have been performed on Table 3.

(Line 179): Table 3. Parameter values of limit states of 750 kV main transformer

| Key vulnerability parameters | Parameter values of limit states | | | |
| --- | --- | --- | --- | --- |
|  | Complete | Extensive | Moderate | Slight |
| $U_{g}$(mm) | 600 | 420 | 240 | 120 |
| $S_{g}$ (MPa) | 40 | 28 | 16 | 8 |
| $U_{z}$ (mm) | 200 | 140 | 80 | 40 |
| $S_{z}$(MPa) | 40 | 28 | 16 | 8 |

**(3.1.2)**

**8:** In Table 4, parameters are not defined clearly, e.g. attribute value 0.7019 refers to characteristic (what)? [may be characteristic period, found later in line 197]

**Response:** 0.70 is natural vibration period of 750 kV main transformer. More specific descriptions of these parameters in Table 4 have been added. At the same time, the criteria by which these parameters are used have been added. (Line 186-188): Based on ‘Code for seismic design of power facilities (GB 50260)’, and ‘Code for seismic design of buildings (GB50011)’, the selection principle for the seismic waves for the IDA of 750 kV main transformer is shown in Table 4.

(Line 189): Table 4. Selection principle of ground motion records

| Parameter | Natural vibration period of 750 kV main transformer (s) | Venue category | Earthquake grouping | Earthquake influence coefficient |
| --- | --- | --- | --- | --- |
| Attribute value | 0.7091 | 3 | 3 | 0.08 |

**9:** It should be clearly mentioned why earthuakes with very low PGA value (0.0180g - 0.0597g) are selected for IDA. What is the type of magnitude of the earthquakes (Mw, Ms...)?

**Response:** Thanks for your suggestions. These seismic waves searched in the PEER strong earthquake database were recorded by seismic stations at some distance from the epicentre. Therefore, their PGA value is relatively small. After the corresponding amplitude modulation, it can be used for numerical calculation. The authors have revised the relevant statements in Table 5 and added Rjb (Joyner-Boore distance) for these seismic stations. The type of magnitude of the earthquakes is M_S._

(Line 194): Table 5. Pieces of information related to ground motion records

| Number | Time and Station Name | Joyner-Boore distance (km) | M_S_ | PGA (g/10^-2^) |
| --- | --- | --- | --- | --- |
| 1 | 1941 Ferndale City Hall | 91 | 6.6 | 4.53 |
| 2 | 1942 El Centro Array | 57 | 6.5 | 2.86 |
| 3 | 1951 El Centro Array | 25 | 5.6 | 5.90 |
| 4 | 1952 LA - Hollywood Stor FF | 115 | 7.36 | 5.33 |
| 5 | 1952 Pasadena - CIT Athenaeum | 123 | 7.36 | 4.48 |
| 6 | 1952 San Luis Obispo | 73 | 6 | 3.71 |
| 7 | 1953 El Centro Array | 15 | 5.5 | 4.39 |
| 8 | 1955 El Centro Array | 14 | 5.4 | 5.17 |
| 9 | 1956 El Centro Array | 121 | 6.8 | 5.14 |
| 10 | 1960 Ferndale City Hall | 57 | 5.7 | 5.97 |
| 11 | 1966 Cholame - Shandon Array | 18 | 6.19 | 4.72 |
| 12 | 1960 San Onofre - So Cal Edison | 129 | 6.63 | 4.15 |
| 13 | 1968 Via Tejon PV | 55 | 6.61 | 2.91 |
| 14 | 1971 LB - Terminal Island | 59 | 6.61 | 3.11 |
| 15 | 1971 Port Hueneme | 69 | 6.61 | 2.38 |
| 16 | 1971 Puddingstone Dam (Abutment) | 53 | 6.61 | 4.55 |
| 17 | 1971 San Juan Capistrano | 108 | 6.61 | 3.99 |
| 18 | 1971 UCSB - Fluid Mech Lab | 124 | 6.61 | 1.80 |
| 19 | 1971 Wheeler Ridge - Ground | 68 | 6.61 | 3.23 |
| 20 | 1971Wrightwood - 6074 Park Dr | 62 | 6.61 | 45.5 |

**(3.1.3)**

**10:** In line 197, it is mentioned that the amplitude adjustment ratios are 0.1g, 0.2g, 0.3g,....,1.5g. Is it amplitude adjustment ratios or amplitudes?

**Response:** We are very sorry for the writing is not clear enough. They are amplitudes not amplitude adjustment ratios. Each seismic wave is modulated into 15 seismic waves with PGA of 0.1-1.5g. The author revised the relevant statements in the article and corrected them.

(Line 208): Table 6. Criterion for earthquake amplitude modulation

| Characteristic period of structure *T*(s) | Amplitude of PGA |
| --- | --- |
| *T* ≤ 0.5 s | {0.1 g, 0.2 g, 0.3 g, …, 3.0 g} |
| 0.5 s < *T* ≤ 2 s | {0.1 g,0.2 g,0.3 g, …, 1.5 g} |
| 2 s < *T* | {0.1 g, 0.2 g, 0.3 g, 0.4 g, 0.5 g} |

**11:** In Fig. 4, what is meant by [Ug/m], [Sg/MPa], [Uz/m], [Sz/m]?

**Response:** We are very sorry for the lack of detail. We have added a description of these parameters in the text. (Line 215-219): These figures indicate that the seismic response values of four key vulnerability parameters ($U_{g}$, $S_{g}$, $U_{z}$, $S_{z}$) under 20 sets of seismic waves, where the red line is the median of the seismic response values of 20 seismic waves under different PGA (0.1 g, 0.2 g, …, 1.5g). The bushing comprises a brittle material, and shows no evident deformation. Therefore, the shaded part in the figure indicates that the bushing has been completely destroyed.

**12:** In Fig. 4c, why >0.2m is marked while the limit is defined as 0.6m (in Fig. 4a >0.6m is marked for same limit state value)?

**Response:** We are very sorry for our incorrect writing. Fig 4c is the seismic response calculation result of displacement at the top of medium-voltage busing (U_Z_). In the unmodified version, the limit value of U_Z_ in Table 3 should be 0.2m, not 0.6m. The authors have made changes to Table 3.

(Line 179): Table 3. Parameter values of limit states of 750 kV main transformer

| Key vulnerability parameters | Parameter values of limit states | | | |
| --- | --- | --- | --- | --- |
|  | Complete | Extensive | Moderate | Slight |
| $U_{g}$(mm) | 600 | 420 | 240 | 120 |
| $S_{g}$ (MPa) | 40 | 28 | 16 | 8 |
| $U_{z}$ (mm) | 200 | 140 | 80 | 40 |
| $S_{z}$(MPa) | 40 | 28 | 16 | 8 |

**(3.1.4)**

**13:** Parameters in equation 4 should be defined clearly.

**Response:** We are very sorry for the lack of rigorous writing. We have added the description of each parameter in Eq. 4. (Line 237-248):

$P\left[D\geq C/IM\right]=\Phi\left(\frac{\ln\left(IM\right)-\frac{\ln\overline{C}-\ln a}{b}}{\frac{\sqrt{\beta_{D/IM}^{2}+\beta_{C}^{2}}}{b}}\right)$ (4)

Here, $\overline{D}$is the median value of the seismic response of structure; $\overline{C}$ is the median value of the structural seismic capacity, i.e., ultimate failure value of structure under earthquake action;$\beta_{C}$is the standard deviation of the structural seismic capacity, in general, take 0.2; $\beta_{D/IM}$ is the log standard deviation of the structural seismic demand, and its median is $\ln\overline{D}$; its standard deviation is $\sigma$. $a$ and $b$ are unknown parameters that must be obtained through regression analysis. The value of $\beta_{D/IM}$, $a$, and $b$ can be estimated as follows:

$\ln\left(\overline{D}\right)=b\ln\left(IM\right)+\ln\left(a\right)$ (5)

$\beta_{D/IM}\cong\sqrt{\frac{\sum\left(\ln\left(d_{i}\right)-\ln(aIM_{i}^{b})\right)^{2}}{N-2}}$ (6)

In the above, $d_{i}$ is the peak earthquake demand, and $IM_{i}$is the peak ground motion. $N$ is the number of samples.

**14:** In line 229, though there is no section as 3.3.2 it mentioned as... simulation results from 3.3.2.

**Response:** We are very sorry for our incorrect writing. It should be 3.1.3, not 3.3.2. We have added relevant content, explaining in detail how the results of IDA analysis in 3.1.3 applied to 3.1.4. (Line 247-249): The seismic response results of each key vulnerability parameter from 3.1.3 was input into Eq. (5) to calculate the $\beta_{D/IM}$ value, as shown in Table 7.

**(3.2.1)**

**15:** There is no description about the experts evaluation. The importance weights in Table 6 (addressed as Table 8 in text) should be checked.

**Response:** We are very sorry for our unclear writing. Power facility classification and zoning and importance weights are cited from the enterprise standard, i.e. ‘Guidelines for seismic safety risk assessment of electrical substations (converter stations)’. This book was compiled by the two presenteers of this article, Shanghai Jiao Tong University and China Electric Power Research Institute. Three authors of the article participated in the compilation process. The author has marked references to the weight of importance in the text. (Line 294-297): This study adopts an importance weight assignment method to construct vulnerability models for substations with different voltage levels. The power facilities are classified based on their structural characteristics, seismic damage characteristics, and functions, as shown in Fig 7. Subsequently, the importance weight of each district power facility is obtained through referring to this criterion [25].

(Line 498-499): [25] State Grid Corporation of China. Guidelines for seismic safety risk assessment of electrical substations (converter stations). Beijing, China: China Electric Power Press. 2021.

**(3.2.2)**

**16:** What parameters are considered for nine different facilities and What are the limit state for those vulnerable parameters for different damage states?

**Response 16:** Due to the limitation of space, the analysis process and results of all power facilities cannot be included in the text. Other different facilities have been submitted to the data availability declaration.

**(4.1)**

**17:** The magnitude of 2008 Wuqia earthquake (Richter M 6.8) should be converted to Ms value as in the attenuation equation (eqn 1), it is denoted that magnitude should be in Ms.

**Response:** China officially uses the surface wave (M_S_) magnitude for earthquakes. All the earthquakes in the text are of surface wave magnitude. Due to an oversight in writing, the problem was not taken into account. The authors have unified the expression of the text and examined the data.

**(4.2.1)**

**18:** In equation 10, N(>=M) denotes, rate of earthquake i.e. number of earthquakes per year with magnitude greater or equal M. This should be described correctly and also must be applied in the development of Magnitude-Frequency relationship.

**Response:** We are very sorry for our unclear writing. Thanks to the expert who pointed out the mistakes in our paper. The previously used method could not get the upper limit of the magnitude of the region, so I used Mabuti Hiroshi’ (2004) method to get the solution again. (Line360-388): The magnitudes and frequencies of earthquakes, whether global or regional earthquakes, generally satisfy Gutenberg-Rick's law, as shown in Eq. (11) [32]. But the model does not take into account the upper limit of magnitude, as shown in Eq. (12) [33]. In 1971, Utsu T proposed a modified G-R model that can take into account the upper limit of magnitude. Therefore, the modified G-R model was used to calculate the upper limit of the magnitude of the Wuqia-Kashgar earthquake.

$\lg n\left(M\right)=a-bM$ (11)

$\lg n\left(M\right)=a-bM+\lg\left(M_{c}-M\right)$ (12)

In the above, $n\left(M\right)$ is the number of earthquakes in the magnitude $M-\Delta M$ to $M+\Delta M$ range. The cumulative frequency is that $N\left(M\right)=\int_{M}^{\infty} n\left(M'\right)dM'$. The value of $a$ describes the level of seismic activity in the selected area, and the value of $b$ is related to the regional medium characteristics, stress state, and inhomogeneity, and can reflect the source $b$ characteristics of the earthquake. $M_{c}$ is the upper limit of regional magnitude.

For parameters $a$ and $b$, this study used the maximum likelihood method given by [33] to estimate their values. In the probability density function $f\left(X\right)$ of this model, $X=M-M_{z}$ ($M_{z}$ is the lower limit of the magnitude of the analysis data) is taken as the probability variable, and is given in Eq. (13):

$f\left(X\right)=B^{2}\left(C-B\right)\exp\left(-BX\right)/P$ (13)

In the above, $B=b\ln\left(10\right)$, $C=M_{c}-M_{z}$, $P=\exp\left(-BC\right)+BC-1$. (14)

Then, the probability density function is expressed by two numbers, $B$ and $C$. If the number of earthquakes contained in a set of data is set as N, the logarithmic likelihood can be calculated by Eq. (15).

$\ln\left(L\right)=N\left(2\ln B-\ln P-B\sum_{i=1}^{N} X_{i}/N\right)+\sum_{i=1}^{N} \ln\left(C-X_{i}\right)$ (15)

In Eq. (14), the conditions for maximizing the logarithmic likelihood are that:

$\frac{\partial}{\partial B}\ln L=0,\text{ }\frac{\partial}{\partial C}\ln L=0$ (16)

Thus, $B$ and $C$ can be obtained from Eq. (15), and (16). After that, $b$ and $M_{c}$ can be further solved by Eq. (14).

The historical seismic information from the last 20 years, as shown in Fig 11(a), was taken as the data sample. The magnitude-cumulative frequency relationship was plotted as a curve, as shown in Fig 14. It can be seen that the cumulative frequency did not change within the magnitude range of 1.0–2.9. Therefore, it could not be included in the calculation range of the model. Between the magnitudes of 2.9, and 6.8, the linear relationship was good and the cumulative frequency distribution decreased with the increase in *M*, indicating that the data was complete and could be used for the model calculations.

(Line 515-516): [33] Mabuti hiroshi yasushi. ôtake masakazu. satô haruo. Distribution of global maximum magnitude Mc based on modified G-R magnitude-frequency distribution model. World Earthquake Translations,2004(01):1-13.

**19:** The completeness of data can be checked by the plot of Cumulative No. of earthquakes Vs Time (Year) as per the simplified approach by Mulargia et al. (1987), or even by the method of Stepp (1972).

**Response:** Considering the reviewers’ suggestion, we have checked the completeness of data. These are all the magnitudes recorded in this region since observations began.

**20:** In the Magnitude-Frequency curve, vertical axis (earthquake frequency) should be plotted in log scale to represent the linear relationship.

**Response:** We are very sorry for our incorrect writing. We have modified the Fig 14. (Line 389):

Fig 14. Magnitude-cumulative frequency curve

**21:** It is not clear why the occurrence rate or frequency of maximum magnitude earthquake is 1, i.e. No. of earthquake per year is 1 [N(>=M)=1].

**Response:** Thanks to the expert for pointing out my mistake. It is wrong to think that the magnitude whose cumulative frequency is 1 is the upper limit for the region. There were errors in the previous method, and now we used a modified G-R model to analyze the upper limit of magnitude in the region. The upper limit of magnitude (M_S_) by using the modified G-R model.

(Line 360-392):The magnitudes and frequencies of earthquakes, whether global or regional earthquakes, generally satisfy Gutenberg-Rick's law, as shown in Eq. (11) [32]. But the model does not take into account the upper limit of magnitude, as shown in Eq. (12) [33]. In 1971, Utsu T proposed a modified G-R model that can take into account the upper limit of magnitude. Therefore, the modified G-R model was used to calculate the upper limit of the magnitude of the Wuqia-Kashgar earthquake.

$\lg n\left(M\right)=a-bM$ (11)

$\lg n\left(M\right)=a-bM+\lg\left(M_{c}-M\right)$ (12)

In the above, $n\left(M\right)$ is the number of earthquakes in the magnitude $M-\Delta M$ to $M+\Delta M$ range. The cumulative frequency is that $N\left(M\right)=\int_{M}^{\infty} n\left(M'\right)dM'$. The value of $a$ describes the level of seismic activity in the selected area, and the value of $b$ is related to the regional medium characteristics, stress state, and inhomogeneity, and can reflect the source $b$ characteristics of the earthquake. $M_{c}$ is the upper limit of regional magnitude.

For parameters $a$ and $b$, this study used the maximum likelihood method given by [33] to estimate their values. In the probability density function $f\left(X\right)$ of this model, $X=M-M_{z}$ ($M_{z}$ is the lower limit of the magnitude of the analysis data) is taken as the probability variable, and is given in Eq. (13):

$f\left(X\right)=B^{2}\left(C-B\right)\exp\left(-BX\right)/P$ (13)

In the above, $B=b\ln\left(10\right)$, $C=M_{c}-M_{z}$, $P=\exp\left(-BC\right)+BC-1$. (14)

Then, the probability density function is expressed by two numbers, $B$ and $C$. If the number of earthquakes contained in a set of data is set as N, the logarithmic likelihood can be calculated by Eq. (15).

$\ln\left(L\right)=N\left(2\ln B-\ln P-B\sum_{i=1}^{N} X_{i}/N\right)+\sum_{i=1}^{N} \ln\left(C-X_{i}\right)$ (15)

In Eq. (14), the conditions for maximizing the logarithmic likelihood are that:

$\frac{\partial}{\partial B}\ln L=0,\text{ }\frac{\partial}{\partial C}\ln L=0$ (16)

Thus, $B$ and $C$ can be obtained from Eq. (15), and (16). After that, $b$ and $M_{c}$ can be further solved by Eq. (14).

The historical seismic information from the last 20 years, as shown in Fig 11(a), was taken as the data sample. The magnitude-cumulative frequency relationship was plotted as a curve, as shown in Fig 14. It can be seen that the cumulative frequency did not change within the magnitude range of 1.0–2.9. Therefore, it could not be included in the calculation range of the model. Between the magnitudes of 2.9, and 6.8, the linear relationship was good and the cumulative frequency distribution decreased with the increase in M_S_, indicating that the data was complete and could be used for the model calculations.

Fig 14. Magnitude-cumulative frequency curve

According to the earthquake catalog data, the maximum likelihood method was used to fit Eq. (11)-Eq. (16) to obtain $M_{c}=7.9$. So, the upper limit of regional magnitude (M_S_) of the Wuqia-Kashgar earthquake is 7.9.
